# Neural network enhanced aging time measurements of diary product remaining with infrared spectroscopy^☆^

**DOI:** 10.1016/j.heliyon.2023.e22039

**Published:** 2023-11-04

**Authors:** Tobias Beck, Bernhard Gatternig, Antonio Delgado

**Affiliations:** Lehrstuhl für Strömungsmechanik, Friedrich-Alexander-Universität Erlangen-Nürnberg, Germany

**Keywords:** *Near infrared*, *Neural network*, *Food processing*

## Abstract

The determination of the drying degree of food residues on surfaces is an important step before efficient cleaning can be achieved. To accomplish this goal, a rapid evaluation based on a neural network and non-invasive measurement technique is introduced. Two common starch-based products and various yogurts from different manufacturers are used as example contaminants to determine the aging time of dried food residue. Near-infrared spectroscopy serves as a modern and fast measurement technique for investigating food compositions. Two analysis methods were compared for processing the measured near-infrared spectral data. The raw data were analyzed using partial least squares regression in conjunction with necessary preprocessing steps. As an alternative method, three different types of neural networks are employed. The aim of this approach is to compensate for the filtering steps before regression, which are typically necessary for multivariate regression. The challenge is to measure three different types of food and obtain a reliable prediction of moisture content in order to draw conclusions about the drying time. The experiments have shown that simple flat neural networks have similar accuracy compared to conventional regression. The use of a convolutional layer in advance demonstrates a significant improvement in prediction compared to other neural networks and even manages to surpass the accuracy of PLS regression. A network with a convolutional layer can also compensate for the sometimes strong variations between food types.

## 1 Introduction

The stage of food fouling and product residues on surfaces are relevant parameters for a following cleaning procedure. To determine the degree of soiling is an important step before proper cleaning parameters like time, usage of chemicals and heat were chosen.

In some cases in food production drying of food is a desirable process. The content of water in food can be an important parameter, when it comes to the storage capability. To increase the capability of storage a small water content should be obtained like for pasta, herbs and spices [1,2].

The drying process depends on the aging time, i.e. the running time starting with the intended or not intended contact of the product (food soiling) on the surface to be cleaned later on during the food treatment process. During the drying process, i.e. aging time there are several physical effects which take part in the results. The Basic principle can be described as diffusion process. The mass flux can be in some cases taken as proportional to the surface and a diffusion coefficient according to Fick's law for instance. The equilibrium which will adjusts over time can be described by the activity coefficient of the air-water-sample-mixture [3]. In case that the substance is developing a skin on the top surface, the modeling of the drying process will be much more challenging. This process is very complex and depends on very much parameters, such as air humidity, temperature, structure and the moisture gradient over the sample thickness [4]. A model which is used for drying is the tin layer equation [5,6] based on Fick's law of diffusion. As much models for drying there are many publications to measure the water content in food [4,7,8]. The measuring using near infrared radiation based on the effect that an emitted spectrum of light stimulate the molecular vibration which leads to the emission of a different spectrum of radiation. Through this difference of emitted and the detected radiation, response several chemical properties of the sample can be investigated [9]. For water a very interesting region is at the wavelengths of 900–1000 nm 1400–1500 nm and 1800–2100 nm [10]. Because water is a very good absorber, its presence or absence is a very good indicator for moisture and very much more properties in food applications [[11], [12], [13], [14], [15], [16]].

The NIR investigation of food properties in the dairy industry are widely spread [[17], [18], [19], [20], [21]]. The composition of raw milk (protein, fat, lactose) for example is investigated by several authors [[22], [23], [24]]. NIR analysis can also use to minor the health of the cows by looking at the composition of the milk [25].

The disadvantage, that the spectral data of milk is very similar to water is in this case a big advantage [26]. Measuring the moisture with NIR is a promising method and fast method especially for dairy products like yoghurt [27] and cheese [[28], [29], [30]].

To get the spectral data into a statistical model for calculating the desired property of a sample there is very often a multi linear regression used [31]. The two mostly used methods are the principal component analyses together with the principal component regression [32,33] and the partial least squares regression [[34], [35], [36]]. In case of analyzing, the spectral Data with samples which are so frequency dependent like water and milk the processing of the data with principal component analysis led to success [37].

The infrared spectroscopy in combination with artificial neural networks (ANN) is a new method, which is used next to the established multi-linear models [38,39]. A big advantage in the ANN modelling of the spectral data is the capability of dealing with non-linear trends [[40], [41], [42]]. A new promising work is published where soil properties predicted by a convolutional neural network using near infrared and visible spectral data [43,44]. Also in classifying and content evaluation with convolutional networks and spectral imaging is subject of recent study [45,46].

There are plenty of methods evaluating the composition of dairy products and in some cases even the temporal changes in certain environmental circumstances were investigated like for cheese as an example. Most of convolutional layer application in this case using classification approaches instead of regression [[47], [48], [49]]. But in the research direction of applications to monitor a kind of aging behavior of dairy products similar to fouling there is no promising infrared application available yet.

This work connects the fast and low cost near infrared spectroscopy with a neural network enhanced evaluation of air-dried food samples. The investigated food samples are two flavors of pudding (vanilla and chocolate) as a starch containing diary product and yoghurt as a fermented diary product in three different fat stages. For this case, two approaches (PLS and ANN) for processing the data were compared to get the best result out of the data. The predicting model should give a good start point for cleaning application.

## 2 Material and methods

The NIR measurement is done with a broadband light source and receiver (Polytec PSS 2220, Polytec GmbH, Waldbronn). The Fiber Measuring Head is mounted on a rack where the tilt of the sensor is controllable. In addition, the distance can be adjusted to obtain the needed stand of distance to the sample. The samples were scaled with a laboratory scale (Kern KB 6500-1 N, Kern & Sohn GmbH, Balingen), which allows synchronous communication with the spectrometer and the Laboratory Computer over a serial interface. A drying closet is used to finally dry down each sample.

Every sample is filled in a Petri dish with 100 mm diameter and 7 mm border height. The petri dishes were scaled empty and freshly filled with the sample. An overview of the samples with the initial moisture is given in Table 1. The content of Fat, Sugar, carbohydrates (CHO) and salt are according to the manufacturers given nutrition table. The Water content is calculated by the drying procedures. Every value is related to a sample of 100 g for better comparison.

**Table 1:** Manufacturer data of nutrition relevant ingredients composition.

Sample	Chocolate Pudding			Vanilla Pudding			Yoghurt		
ID	1	2	3	4	5	6	7	8	9
Fat	9.3	8.2	3.1	8.8	3.3	3.1	0.1	1.5	3.5
Sugar	13.0	13.0	14.0	13.0	12.0	12.9	4.2	4.2	4.1
CHO	3.0	3.0	3.6	2.0	3.0	3.6	0	0	0
Protein	3.3	3.3	3.2	3.0	3.2	3.0	5.4	5.3	4.5
Salt	0.12	0.18	0.13	0.2	0.14	0.14	0.13	0.14	0.13

The samples were prepared in sets of three dishes per drying span and 12 samples per product over the process time of several hours. For each product, this results in a total number of samples of 36 dishes. By clustering each product to the type of diary product, for each type there were 102 samples.

The Water content of every sample is measured be the weight at three different times. The first measurement is at the initial moisture $M_{init}$, which is calculated with equation (1) with the fresh sample ($m_{0}$) and the totally dried sample ($m_{2})$. After the scheduled drying time at the ambient conditions the weight is measured the second time ($m_{1}$). At this stage, the moisture is also measured with the NIR spectrometer. The sample is put in the drying chamber and is measured finally after 48h drying time under 100 °C in the drying chamber and scaled again The initial water content is calculated by the difference of the first and the last measurement in relation to the initial weight.

1 $$M_{init}=\frac{m_{0}–m_{2}}{m_{0}}100\%$$

The moisture $M_{airdry}$ of the sample after the ambient drying is calculated with equation (2).

2 $$M_{airdry}=\frac{m_{0}–m_{1}}{m_{0}}100\%$$

The absorbance spectrum provides information on composition of the sample. It is measured by laying up to a minimum of five data points across the sample surface. The light cone of the spectrometer has a diameter of 5 mm, so a diameter of 100 mm of the Petri dish can provide enough space for the measuring grid and distance between every measuring point without intersection. The data points for each dish are averaged before the data preprocessing. A total Dataset of spectral sampling points of 256 leads after the averaging to a data set of 51 measurements per food sample (in total 459 for all three kinds of food sample). A summary of the generated data is shown in Table 2.

**Table 2:** Overview of the measurement data obtained for the six different food samples and respectively three food types.

Sample ID	1	2	3	4	5	6	7	8	9
Measurements per dish	5	5	5	5	5	5	5	5	5
Measurements per time step	3	3	3	3	3	3	3	3	3
Measurements over time	17	17	17	17	17	17	17	17	17
Measurements in total	255	255	255	255	255	255	255	255	255
After averaging per dish	51	51	51	51	51	51	51	51	51
Per food sample	153			153			153		

A common procedure in processing spectral data with the Partial Least Squares (PLS) method or Principal Component Analysis (PCA) regression is to pre-process the data using filter algorithms. In this work two commonly used algorithms were used to suppress the high frequency noise and the low frequency noise. To compensate variation in the spectra due to surface inhomogeneity for example [50], the first processing step is the normalization of the data using the Standard Nominal Variate (SNV) method. The SNV (equation (3)) subtracts the mean value of the spectrum ${\bar{X}}_{i}$ from the consideration single data point and references it to the spread of the spectrum around the mean value $\sigma \left(X_{i}\right)$.

3 $$X_{ij}\left(SNV\right)=\frac{X_{ij}–{\bar{X}}_{i}}{\sigma \left(X_{i}\right)}$$

The high frequency noise is smoothed out with a Savitzsky-Golay-Filter with a window size of seven and a third order polynomial [51]. The low frequency noise distortion is compensated by a Baseline correction algorithm performed by subtracting a baseline which is iteratively estimated with a polynomic function of third order [52,53]. The iteration is performed until a certain convergence limit of the parameters is reached. The processed spectra than is forwarded to the regression algorithms.

### 2.1 Multi-variant regression

To analyze the spectral data, the dataset is processed with a multi-linear regression. The basic assumption, which is expressed in equation (4) is, that the moisture for every measurement in the observed range can be expressed as a Model b with the Matrix of the input values X, which contains every spectral data according to the observed sample and noise.

4 $$Y_{moisture}=X_{spectral_{data}}b_{model}+E$$

The dimensions of the matrix in equation (4), are 1 for $Y_{moisture}$ for the left hand side of the equation, because of the single value which is set in correlation to the spectral data, and 3 for the rows in $X_{spectral_{data}}$, according to the number of samples which were taken. For the Spectral Data the columns represents every absorbance for the observed wavelength. The range for n is between 1100 nm and 2200 nm with a step size of 4.27 nm. This leads to a resolution of n = 256.

The algorithm of the PLS regression for this application, where only a one dimensional reference analytic is targeted, can be found in the literature as PLS1 [35]. The PLS algorithm can be implemented by using the Algorithm of Helland [54].

For training the model, the whole dataset is split into a training dataset and a validation dataset via cross validation. A ratio of 25 % is used to the trainings dataset in relation to the validation dataset.

The models quality is measured with the error between the predicted data $y_{pred}$ from the model and the measured data $y_{meas}$ from the experiment. The quality of the trainings- and the validation dataset is measured the same way. According to this the error function for the trainings set is the described equation (5).

5 $$RMSEC=\sqrt{\frac{1}{N}\sum_{i=0}^{N-1}{\left(y_{pred}–y_{meas}\right)}^{2}}$$

Equation (6) expresses the corresponding error for the validation dataset

6 $$RMSEV=\sqrt{\frac{1}{N}\sum_{i=0}^{N-1}{\left(y_{pred}–y_{meas}\right)}^{2}}$$

As a second metric for the evaluation of the models quality the determination coefficient for the predication is calculated.

7 R2=1−∑i=0N−1ymeasi−ypredi2∑i=0N−1ymeasi-ymeas2

Multivariate regression maps multiple independent variables on less dependent variables. Therefor the R-squared estimate suffers from a bias. To correct this bias it is often suggested, that for multilinear application an adjusted version of R^2^ should be used [55,56]. This value is lower than the R^2^ from equation (7) but does not over estimates that much for this use case. The adjusted $R_{a}^{2}$ can be calculated as

8 $$R_{a}^{2}=1-\frac{N-1}{N-p-1}\left(1-R^{2}\right)$$

In equation (8) N gives the number of total observations and p is the number of model parameters or latent variables.

#### 2.1.1 Flat neural network approach

**Fig. 1:**
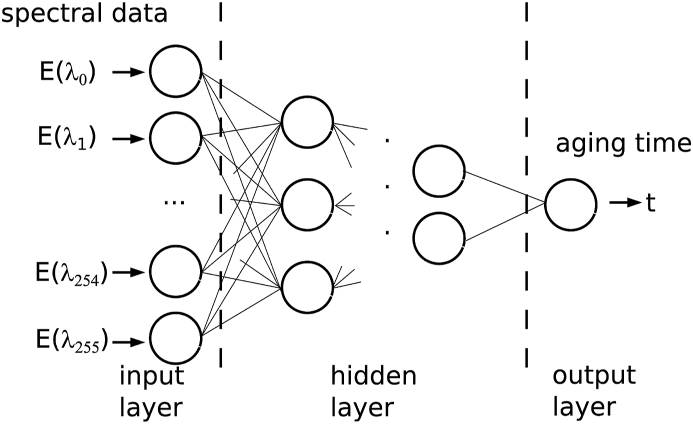
Setup of the artificial neural network in the scheme of a feed forward dense layered network.

The non-linear modelling of the spectral data is built upon an artificial neural network. Neural networks can fit regression tasks as well as statistical models [57]. An artificial neural network is a network of multiple single basic functions with adjustable internal weights, see Fig. 1. Every node just processes the input it gets from various previous nodes and composes them with the internal weights and optional activation functions to an output. This output serves as an input for following nodes which in sum are forming the network. A node can has many inputs (and the same number of internal weights), but only one output. There are three types of nodes used in the network. Input nodes, which have only one input and no previous nodes, output nodes which have no following nodes and the nodes of the hidden layers, which have following and previous nodes and can have multiple inputs. Nodes are clustered in layers. The hidden layers are organized between the output and the input layer. The activation function and the weight of every input line of a node affects the output. The output O is in this case a linear combination of the weights $w_{i}$ and the inputs $I_{i}$, which passing through an activation function φlikeitisdescribedinequation9. The activation function serves as a dumping filter, to trigger the neuronal node not for every input signal like noise. The weights are the main adjustable parameter in the network and in every neuron.

9 $$O=\sum_{i=0}^{N}\varphi \left(I_{i}\right)\;w_{j}$$

The network consists of an input layer which has the amount of nodes according to the number of sampling points from the spectroscope (256). The layer is organized as a regression network. For sake of convenience in the considered case of drying the output layer consist only of one single node for the moisture or drying time. The input layer, on the other hand, consists of 256 nodes for all investigated networks in the following, which forward the individual measuring points corresponding to wavelengths E ($\lambda_{i}$) of the spectrum into the network.

The network is structured with fully connected layers, which means, that every node is connected to every node in the following layer. The estimation of the hidden layer nodes is based on different publications [58,59]. The number of necessary hidden layers is investigated by trial and error, by increasing the number of neurons in the hidden layer each step. The error of the prediction serves as a metric to find the number of nodes that appears appropriate.

##### 2.1.1.1 Convolutional network approach

**Fig. 2:**
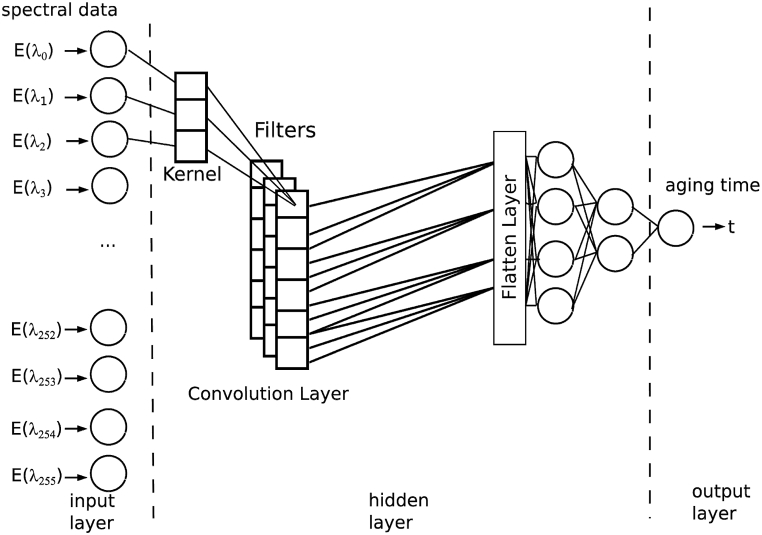
Setup of the artificial neural network with a convolutional layer before the dense layer.

A second approach to process the data using a neural network is to use convolutional layers. In convolutional layers a kernel is convoluted over the neural nodes and creates the output (see Fig. 2). A kernel in this context is a discrete vector of weights. Other as the neural network technique described in the previous chapter it doesn't get linear combined, but convoluted like it is expressed in equation (10). In this equation the sum is built over the number of weights in the kernel $N_{W}$ and the number of Inputs $N_{i}$ of the previous layer [60].

10 $$O=\left(\vec{I}*\vec{w}\right)=\sum_{i=0}^{N_{i}}\sum_{k=0}^{N_{W}}\varphi \left(I_{i}\right)w_{k}$$

The parameters of the convolution kernel are the adjustable parameters like the weights of the flat layer. The convolutional layer brings the effort, that it can be setup using very much lower amount of adjustable parameters and operational complexity [61].

Applying a convolutional neural network can do the preprocessing steps, which are necessary for the calculation of the principal component based regressions within the convolution layer [62].

##### 2.1.1.2 General design and training procedure

The Data for the network were also split according to cross validation into a trainings set and a validation set. The metrics for the model quality were the same as for the multi linear models in equations 4, 5, 6.

As activation function in this case is the rectified linear unit function (ReLU) is used to speed up the learning convergence [63]. The activation function φ like it is described in equation (11) it suppresses every input I with a negative value and damps the non-negative inputs with an factor a.

11 $$\varphi \left(I\right)=\left\{ \begin{array}{c} aI\;\;\;\;\;if\;I>0\\ 0\;\;\;\;\;\;if\;I<0 \end{array}\right.$$

For each training the initial weights are distributed in a Gaussian distribution, with a mean of zero standard deviation σ which is expressed by equation (12) where N is the number of nodes in one layer [64].

12 $$\sigma =\sqrt{\frac{2}{N}}$$

The adjustment process of the weights is performed by back propagating the prediction error back through the network. The basic principle of adjusting is by using the error function with the gradient decent method like it is described in equation (13) where the weights of layer i and node j at the current learning cycle t are set to a relationship with the gradient of the error function. The parameter η serves as an adjustable parameter, which is defined by the used training method.

13 $$w_{ij,t+1}=w_{ij,t}-\eta \frac{\partial \left(Error\;Function\right)}{\partial w_{ij}}$$

For increasing the convergence speed of the training, the network is optimized by using the adaptive momentum estimator (ADAM) [65]. The Optimizer combines estimates of the gradients to adaptively adjust learning rates during training. The weights are updated using the adaptive learning rates derived from the first and second moment estimates of the gradients to guide the direction of parameter updates.

The training is performed by dividing the trainings set in batches. Each fully performed run with all batches counts as epoch, which is equal to a whole cycle of data passing through the network. The Training consists of at least 100 epoch.

The computational part of the work is programmed in python. For the statistical and linear regression part, methods from the packages NumPy 1.26.6, scikit-learn 1.0.1 where used. The neural network is programmed based on the packages of Keras 2.8.0 and TensorFlow 2.8.0. The visualization is done with matplotlib 3.1.1. The programming is done on a windows 10 laptop with the python 3.7.3 environment.

## 3 Results and discussion

The initial moisture of each sample gives information of the total water content of the samples. The results are written in Table 3. For the pudding samples the total content of water is in a range of 65–78 %. The results for the yoghurt lays in a range of 85 to up to 89 %. The moisture of yoghurt in comparison to the pudding is more evenly distributed. The pudding spreads over the different manufactures in a much higher range.

**Table 3:** Content of each sample according to the manufacturer's nutrition table and the measured moisture content.

Sample	Chocolate Pudding			Vanilla Pudding			Yoghurt		
ID	1	2	3	4	5	6	7	8	9
Manufacturer declaration	28.72	27.68	24.03	27.0	21.64	22.74	9.83	11.14	12.23
Water	64.6 ± 2.2	71.4 ± 0.1	78.2 ± 0.3	68.5 ± 1.5	77.2 ± 0.5	78.1 ± 0.2	89.4 ± 0.5	85.9 ± 0.3	85.6 ± 0.9
Other	6.68	0.92	−2.23	4.5	1.16	−0.84	0.77	2.96	2.17

According to the declared content of CHO, sugar, fat, protein and salt the measured water content shows except of two samples a rest of non-defined other content. For sample 1 and 6 the residual value of the remaining non declared or measured content is negative and indicates an error in the water measurement or the manufactures declaration. Respectively to the total amount of other values it will further be neglected.

**Fig. 3:**
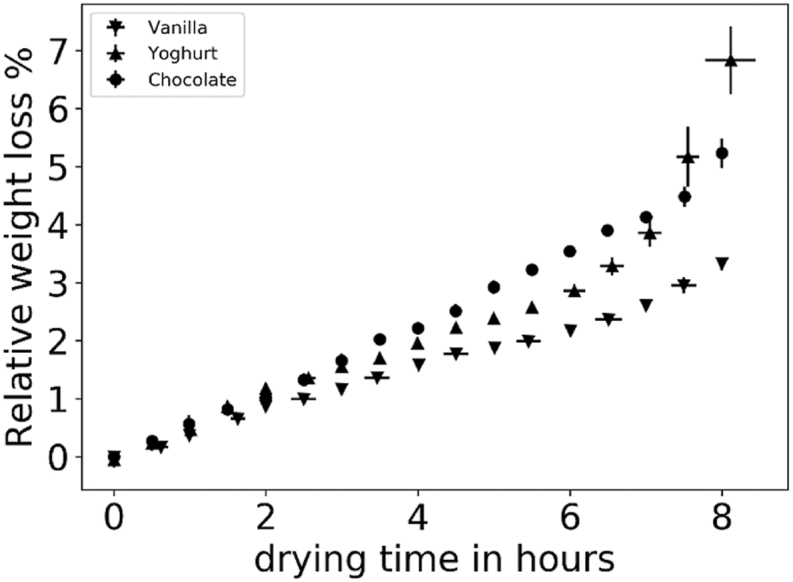
Relative weight loss of chocolate and vanilla flavored pudding and yoghurt over a drying period of 8 h.

The samples are weighted after every spectral measurement and then dried completely down and weighted again. The water content from the spectral measurement is given by equation (1), the total water content of the sample is given in relation to the fresh sample. Over the process time the samples are scaled at every time it is measured by the near infrared device. To calculate the weight loss at air drying the relative weight loss according to equation (2) is recorded. The weight loss over the time is displayed in Fig. 3.

Each sample shows a nearly similar progress in the first hours of drying, which is nearly linear. After the half of the drying time, the differences between the drying behaviors are more visible. Where yoghurt shows a stronger increasing water loss, the pudding samples differs in values but are showing a similar slope. As a result of the reference analytics it can assumed, that for samples younger than 4 h, there can be used a direct proportional correlation between drying time and water loss. Since the water content is a central parameter that can be measured using near-infrared spectroscopy, this finding represents an important basis for further work. Where the weight loss is the resulting value due to drying, the time serves as the aim for the regression.

**Fig. 4:**
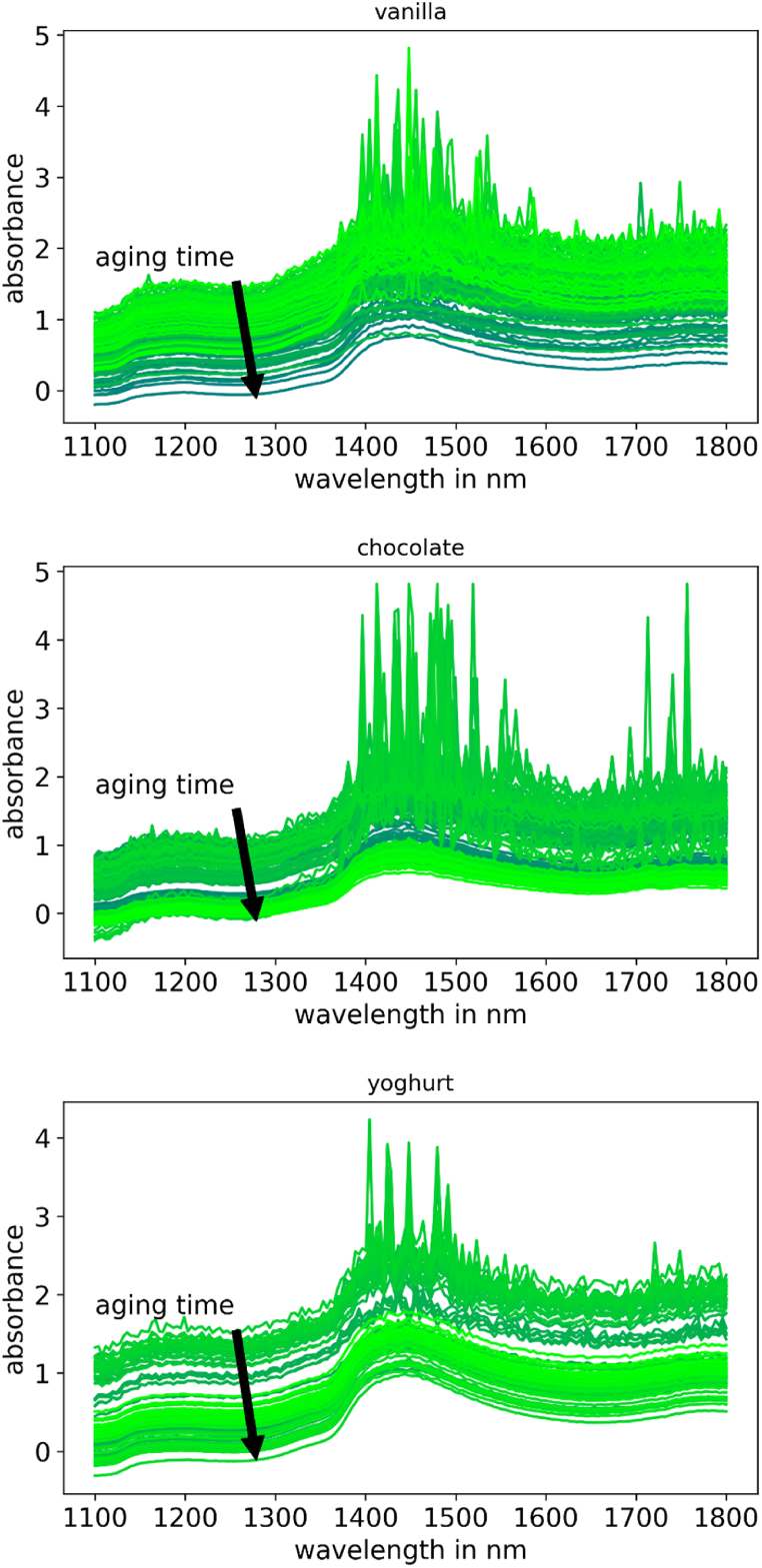
unprocessed spectral data for the different food samples. From left to right: vanilla flavored pudding, chocolate pudding and native yoghurt.

**Fig. 5:**
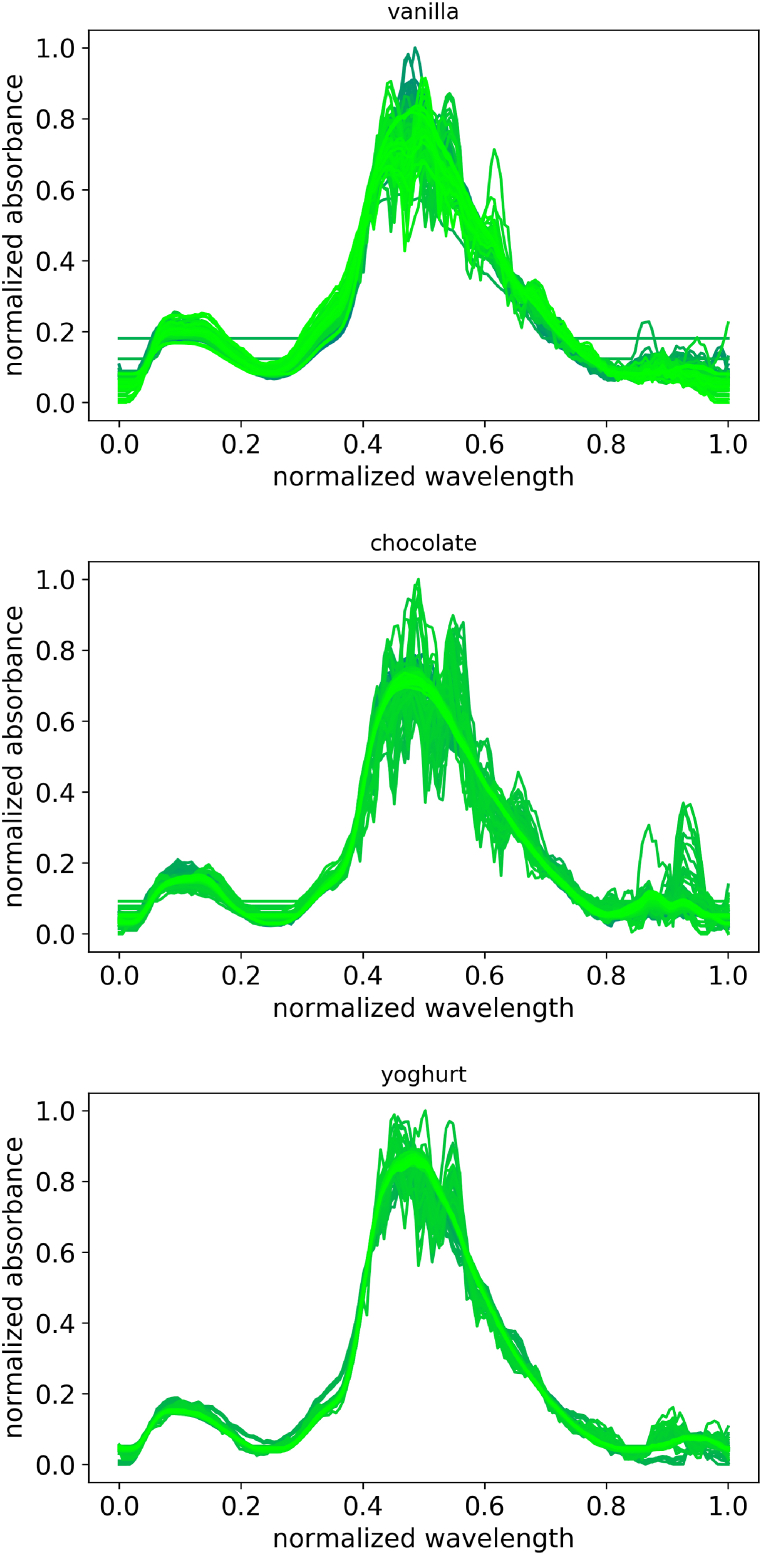
processed data for each sample. Processing is performed by smoothing, baseline correction and normalization according to mean value and standard variation of each spectra.

The very basic results of the spectrometric measurement shows a typical course for food samples under near infrared. The absorbance is for each dish averaged displayed in Fig. 4. It can be recognized that the typical water peak around 1300 nm and in the range of 1600–1800 nm are detected in every sample. The raw spectral data shows very much noise especially in the higher frequency ranges. Also every sample shows a dc shift or a baseline deviation, which is later normalized out to get comparable spectral data. For processing the data with PLS, a three step preprocessing routine is performed. At first, the values were smoothed with a Savitzky-Golay filter followed by a baseline correction. The baseline is iteratively calculated for each spectrum with a fifth order polynomic function by the algorithm explained in. The filtered spectral which are displayed in Fig. 5 are showing a much better comparable data. The Standardization, which is used for the multivariate regression is done with the standard normal variate method. To filter out the high-frequency noise, a Savitzky-Golay filter with a third-degree polynomial and a window width of seven has been used. The baseline is smoothed using a third-order polynomial function. For the neural algorithm the data is just normalized between zero and one according to the minimal and maximal values of the whole data set.

The processed spectral data are showing the same location of the peaks as the raw spectra, but without that many high fluctuation noise and the low fluctuate noise which leads to the increasing course at the high wavelengths. The different lines are still too close to each other which doesn't allow a decent regression and prediction that makes the analysis of the multivariate data necessary. Where the aging time can in some spectral data as it is shown in the annotations in Fig. 4 can be estimated through the position of the curve in the plot, it shows even curves, which do not follow this first assumption. It is also noticeable, that the fresher the sample is, the noisier it gets. Especially vanilla pudding shows this effect, if a light green curve is compared to a darker one. So it can be derived, that the presence of water correlates in some ways with high fluctuating peaks in the measured signal. In the processed data for the regression, there can be no aging time derived only through the position of the curve in the bulk (Fig. 5).

For each model, the original dataset, consisting of the measurement values from reference analytics ($Y_{moisture}$) and their corresponding spectral data ($X_{spectral_{data}}$), is randomly split into a calibration and validation dataset in a 75 %–25 % ratio.

### 3.1 Partial least squares regression

**Fig. 6:**
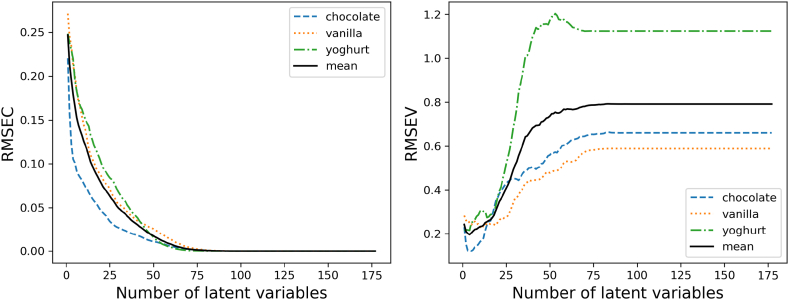
Error (left) and r-squared values (right) for the PLS Regression over the number of latent variables as adjustable parameters.

As in the previous chapter described Partial least squares regression is based on the method of building latent variables from the dataset of the independent and setting them in relation to the dependent variables. The number of necessary latent variables or components is investigated by iterating over the datasets and increasing the number of components until a satisfying convergence level of the models metrics are reached. In this case the model metrics are the root mean squared errors of the model. The r-squared suffers in multi-variant data regression as previously proposed from an overestimation of the explained correlation, so it can be adjusted which results in a lower value. Due to this, this metric is only used for comparison. The error of the regression according to a variable is shown in Fig. 6.

A minimal error value in the calibration is reached after 75 latent variables. In comparison to the calibration, the validation is showing minimal values only under a latent variable count of 25. A closer look and evaluating additional the r-squared value shows the optimal value for all three samples cannot be achieved. In Table 4 are the best performances for the three types of food soiling listed.

**Table 4:** RMSE und R^2^ comparison of the three food samples.

	chocolate n = 5		vanilla n = 11		yoghurt = 4	
	calibration	validation	calibration	validation	calibration	validation
RMSE	0.101	0.121	0.138	0.226	0.219	0.217
R^2^	0.891	0.843	0.790	0.468	0.36	0.36

Especially the model for yoghurt shows a very poor accuracy in this case. Chocolate pudding has with 5 latent variables the best fitted model. The vanilla model shows an indication of overfitting with the large difference between the calibration and validation metrics. The vanilla model shows with the great difference between the calibration and validation metrics an.

The PLS Regression is taken as a base for assessing the performance of the neural regression approaches. The three different approaches of neural network regression are discussed for each of the approaches in the following. In general, the layers of the networks are designed similarly. The Rectified Linear Unit (ReLU) function is defined for all inputs of the individual neurons. The initialization of the weights is done by a Gaussian distribution with a standard deviation proportional to the reciprocal number of nodes in the layer.

#### 3.1.1 Artificial neural network

**Fig. 7:**
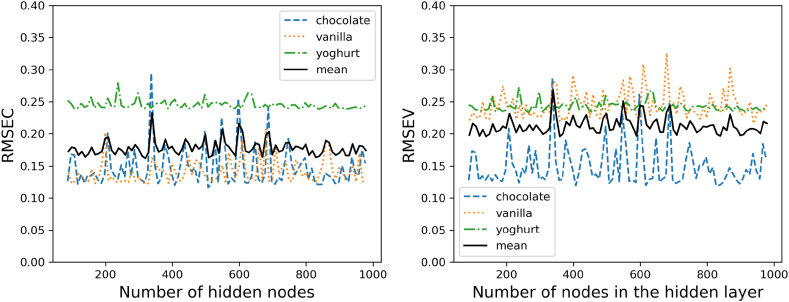
ANN performance error for calibration (left) and validation (right) over the varying number of hidden nodes in the hidden layer.

The simplest neural network-based regressor considered is a three-layer network consisting of an input layer, a hidden layer and an output layer. This neural network will be referred to as ANN in the following. The optimal number of parameters, respectively hidden nodes are evaluated by increasing their number, and comparing the networks performance. As a performance metric there is also the root mean squared error and the r-squared used. The errors over the number of parameters are displayed in (see Fig. 7).

The progression of the errors over the various changed parameters shows a very irregular progression. The mean values of the individual samples show no clear minimum over the course of 100–1000 hidden nodes. While the two types of pudding show a similar calibration curve, the error for yoghurt is comparatively high. Similar results were shown for the previous PLS model. The course of the validation of the model shows a constantly high error of the model for yoghurt as well as higher values for vanilla pudding. Chocolate pudding remains similarly low as in the calibration.

#### 3.1.2 Deep neural network

The second approach represents a neural network that supports two additional hidden layers compared to the previous model. The number of individual nodes was also determined by varying the number and then comparing the error values among the different models.

**Fig. 8:**
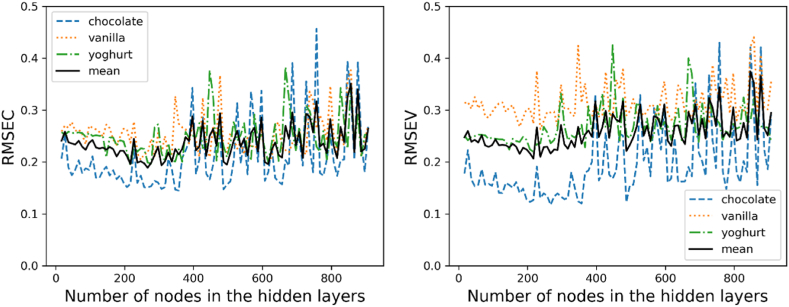
Deep neural network performance error for calibration (left) and validation (right) over the varying number of nodes in the three hidden layers.

This improved version of the three-layer neural network shows a similarly irregular course. However, both the calibration and the validation show a local minimum in a range around 200 nodes per layer. This also becomes clear when all three food samples are averaged. However, a clear optimum even for a range of parameters is not recognizable for any of the sample and node numbers (see Fig. 8).

#### 3.1.3 Convolutional neural network

The third approach, which is varying from the previously discussed two is using a layer of convolutional filters before a dense layer of hidden neurons before the output layer. The adjusted parameter for evaluating the optimal number layer setup in this case are the number of filters used. The Kernel which is the size of each filter is equally chosen as fife elements in size. The number of filters is changed in a range of 2–18 convolutional filters. Due to the higher computing power that a convolutional layer has compared to a normal flat dense layer, only nine different network and filter sizes were calculated for the CNN. The convolutional network starts with an input layer, like the previous discussed models. Following this layer, there is a variable-sized convolutional layer that is adjustable to determine the optimal number of filters. After the convolutional layer, there is a layer that translates the results of the convolutions into a flat layer. A dense flat layer with 200–1000 nodes, similar to previous network models, is inserted, before going into the output layer. The activation function for each information-processing node is also the ReLU function.

**Fig. 9:**
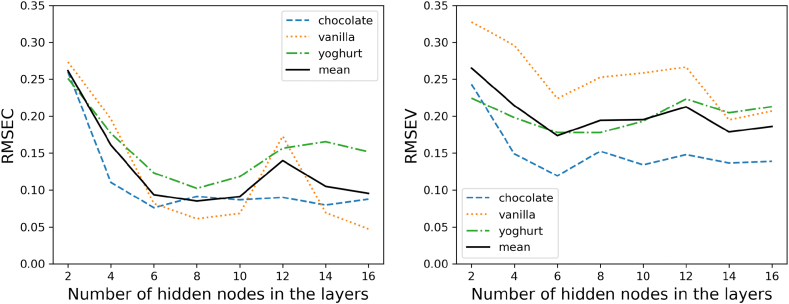
Convolutional neural network performance error for calibration (left) and validation (right) over the varying number of filters in the convolutional layer.

All in all, the result is much less complex than the two previous approaches (Fig. 9). The minimum in the parameter range considered is 8 filters for the calibration and six filters for the validation. This also becomes clear when looking at the average error curve of the three samples. What is striking in comparison to the previous approaches, including the partial least squares model, is the significantly better performance of the model for yoghurt. Although this is still below the accuracy of the other two samples in the validation, it is overall lower than for the partial least squares model.

A closer look at the errors of the model for the six and eight filters shows slight differences between the two models, which is listed in Table 5. Since the difference in calibration between six and eight filters is also not very pronounced, a convolutional neural network approach with six filters can predict the samples with significantly higher accuracy than the other approaches.

**Table 5:** Comparison of the errors of the CNN model for two different filter sizes for calibration and validation.

	chocolate		vanilla		yoghurt	
	calibration	validation	calibration	validation	calibration	validation
6 filters	0.076	0.119	0.081	0.223	0.123	0.178
8 filters	0.091	0.153	0.061	0.252	0.102	0.178

##### 3.1.3.1 Comparison of the approaches

**Fig. 10:**
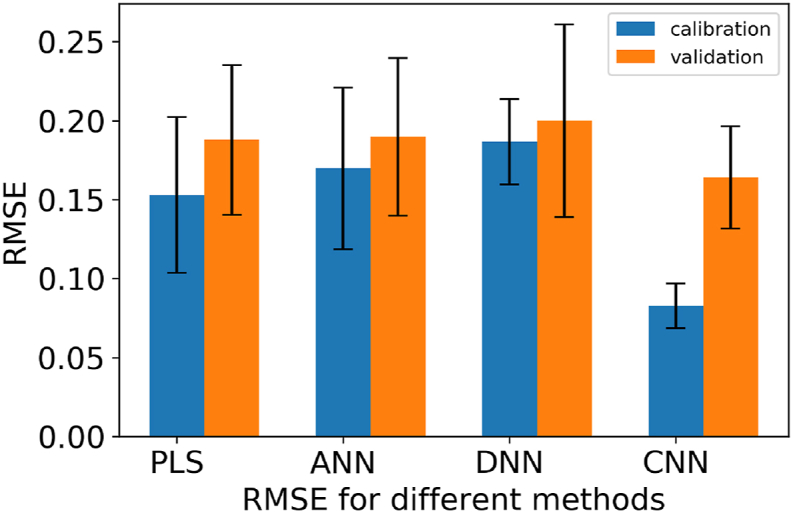
average of the root mean squared error for the different methods with the standard deviation calculated over the different samples.

To figure out the performance of each regression method, the metrics of each sample is compared. The error of the method is represented by the mean of the single error values of each sample type. In Fig. 10 the error values and the errors variance is displayed for the different methods.

What can be derived from this image is, that the network with only one hidden layer and the deep neural network perform in the range of the PLS regression. They have a bit higher errors in the validation of the regression and scatter more around the mean value. In comparison, the approach with the convolutional neural network shows a significantly better overall result with less scatter in the validation. Overall, the validation is less than with the other methods, but also relatively high.

**Fig. 11:**
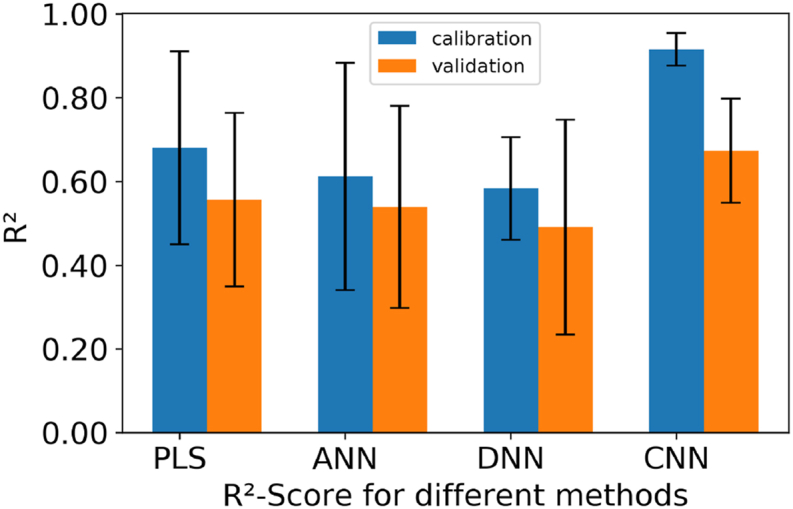
average of the R^2^ score for the different methods with the standard deviation calculated over the different samples.

In order to further consider the quality of the prediction, the R^2^ score is also considered in addition to the RMSE. In Fig. 11 the mean values over the individual samples of the R^2^-scores are shown for the different regression approaches. It can be seen that the neural regressor approaches with one or three hidden layers also perform in the PLS regression range. The mean R^2^-scores are around 0.60 while the spread ranges from 0.40 to 0.90. The scatter shows that they are not uniformly good at predicting sufficiently well for the different foods. The R^2^-score of the CNN on the other side, as it has a score above 0.60 for calibration and validation. In comparison to the scatter ranges, this model approach also has a spread of ±0.05 for the training data set and ±0.1 for the validation. The errors of calibration and the errors of validation and the r-squared lays between 0.72 and 0.90 which is in the range of other work i.e. for measuring the non-solid-fat content of butter with near infrared spectroscopy [13]. The resulting performance range of the neural approach also fits in the results of other fast near-infrared measurements combined with artificial neural networks [66]. In contrast to other publications that measure the content of a specific ingredient in a sample [13,15,28], the approach in this paper is investigating one class of food, with three representatives from the dairy sector and is widely used.

## 4 Conclusion

Near-infrared spectroscopy is often proposed as a fast way of measuring sample compositions. From drying the samples and scaling it can be derived that for fresh samples up to 4 h of drying a linear correlation between drying time and water content can be assumed. Based on this result the water sensible measuring technique of near infrared spectroscopy is can be used to predict the drying time of food soiling. Where partial least squares regression is in many cases the standard for processing spectral data, it can be shown, that artificial neural networks can also predict with a comparable precision. The challenging task of predicting the drying time for three different food samples can also be predicted using a conventional approach. Furthermore, the results of the neural approaches, without the preprocessing required in the PLS approach, demonstrate a comparable quality. A significant enhancement within the neural models presented is achieved by incorporating a convolutional layer. In comparing the four models, the CNN approach stands out with the highest R^2^ values for both training and validation datasets (0.90 and 0.65), showing the best predictive performance. Additionally, the trained CNN shows the lowest RMSE for both training (RMSEC: 0.09) and validation (RMSEV: 0.15), further confirming its effectiveness in accurately modeling the data. For the other two neural network models, the R^2^ ranges for training (around 0.60) and validation (0.50–0.60) suggest reasonably good fits, although they show slightly higher RMSE values compared to PLS Model. Comparing the PLS Regression with the neural networks, it's evident that the regression using CNN outperforms all others, demonstrating the advantages of utilizing a neural network approach in predictive accuracy. The regression with the network approach shows a good adaptability between the three kinds of food in this study. Nevertheless, this comparability can also be attributed to the similar composition with milk as the main component. The adaptability of the network to other types of food or other types of substances in general represents a starting point for future research. Since the aim of the work is to determine the degree of dryness before a cleaning routine, there can be also asked the question how well this described measurement methods and the trained regression procedures perform for online measurement during cleaning. Especially when during the cleaning the dried food soiling is gaining moisture again.

## Funding

This IGF Project of the 10.13039/501100008465FEI is funded via AiF (21507 N) within the program for promoting the Industrial Collective Research (IGF) of the Federal Ministry for Economic Affairs and Climate Action (BMWK), based on a resolution of the German Parliament.

## Data availability

The data that support the findings of this study are available from the corresponding author, TB, on request.

## CRediT authorship contribution statement

**Tobias Beck:** Software, Visualization, Writing – original draft. **Bernhard Gatternig:** Writing - review & editing. **Antonio Delgado:** Writing – review & editing.

## Declaration of competing interest

The authors declare the following financial interests/personal relationships which may be considered as potential competing interests.

## References

[1] Bonaui C. (1996). *u. a.*, „Food drying and dewatering. Dry. Technol., Bd..

[2] Groß F., Benning R., Bindrich U., Franke K., Heinz V. (2011). Und A. Delgado, „Optical online measurement technique used for process control of the drying step during pasta production. Procedia Food Sci., Bd..

[3] Mathlouthi M. (2001). Water content, water activity, water structure and the stability of foodstuffs. Food Control, Bd.

[4] Srikiatden J., Roberts J.S. (2007). Moisture transfer in solid food materials: a review of mechanisms, models, and measurements. Int. J. Food Prop., Bd.

[5] Doymaz I. (2004). Convective air drying characteristics of thin layer carrots. J. Food Eng., Bd.

[6] Özdemir M., Devres Y.O. (1999). The thin layer drying characteristics of hazelnuts during roasting. J. Food Eng., Bd.

[7] Büning-Pfaue H. (2003). Analysis of water in food by near infrared spectroscopy. Food Chem., Bd.

[8] Karmas E. (1980).

[9] Burns D.A., Ciurczak E.W. (2007).

[10] Carleer M. (1999). The near infrared, visible, and near ultraviolet overtone spectrum of water. J. Chem. Phys., Bd..

[11] Roy S., Anantheswaran R.C., Shenk J.S., Westerhaus M.O., Beelman R.B. (1993). Determination of moisture content of mushrooms by Vis—NIR spectroscopy. J. Sci. Food Agric., Bd..

[12] Rodriguez-Otero J.L., Hermida M. (1997). Und J. Centeno, „Analysis of dairy products by near-infrared spectroscopy: a review. J. Agric. Food Chem., Bd..

[13] Hermida M., Gonzalez J.M., Sanchez M. (2001). Und J. L. Rodriguez-Otero, „Moisture, solids-non-fat and fat analysis in butter by near infrared spectroscopy. Int. Dairy J., Bd..

[14] Lestander T.A., Rhén C. (2005). Multivariate NIR spectroscopy models for moisture, ash and calorific content in biofuels using bi-orthogonal partial least squares regression. Analyst, Bd..

[15] Iqbal A., Sun D.-W. (2013). Und P. Allen, „Prediction of moisture, color and pH in cooked, pre-sliced Turkey hams by NIR hyperspectral imaging system. J. Food Eng., Bd..

[16] Alander J., Bochko V., Martinkauppi B., Saranwong S., Mantere T. (2013). A review of optical Nondestructive visual and near-infrared methods for food quality and safety. Int. J. Spectrosc., Bd. 2013, März.

[17] Huang H., Yu H., Xu H. (2008). Und Y. Ying, „Near infrared spectroscopy for on/in-line monitoring of quality in foods and beverages: a review. J. Food Eng., Bd..

[18] Peirs A., Scheerlinck N., Touchant K. (2002). Und B. M. Nicolaï, PH—postharvest technology: comparison of fourier transform and dispersive near-infrared reflectance spectroscopy for apple quality measurements. Biosyst. Eng., Bd..

[19] Kumaravelu C., Gopal A. (2015). Detection and quantification of adulteration in honey through near infrared spectroscopy. Int. J. Food Prop., Bd.

[20] R\uužičková J., Šustová K. (2006). Determination of selected parameters of quality of the dairy products by NIR spectroscopy. Czech J. Food Sci., Bd.

[21] Pu Y.-Y., O'Donnell C., Tobin J.T., O'Shea N. (2020). Review of near-infrared spectroscopy as a process analytical technology for real-time product monitoring in dairy processing. Int. Dairy J., Bd.

[22] Aernouts B., Polshin E., Lammertyn J., Saeys W. (2011). Visible and near-infrared spectroscopic analysis of raw milk for cow health monitoring: reflectance or transmittance?. J. Dairy Sci., Bd..

[23] Sato T. (1987). *u. a.*, „Analysis of milk constituents by the near infrared spectrophotometric method. Jpn J Zootech Sci, Bd..

[24] Šašić S., Ozaki Y. (2001). Short-wave near-infrared spectroscopy of biological fluids. 1. Quantitative analysis of fat, protein, and lactose in raw milk by partial least-squares regression and band assignment. Anal. Chem., Bd.

[25] Aernouts B., Van Beers R., Watté R., Huybrechts T., Lammertyn J., Saeys W. (2015). Visible and near-infrared bulk optical properties of raw milk. J. Dairy Sci., Bd..

[26] Frankhuizen R., Van der Veen N.G. (1985). Determination of major and minor constituents in milk powders and cheese by near infra-red reflectance spectroscopy. Neth. Milk Dairy J. Neth..

[27] Adam M., Dobiáš P., Bajerová P. (2009). Und K. Ventura, „Comparison of various methods for determination of water in white yoghurts. Food Chem., Bd..

[28] Adamopoulos K.G., Goula A.M. (2001). Und H. J. Petropakis, „Quality control during processing of feta cheese—NIR application. J. Food Compos. Anal., Bd..

[29] Frank J.F., Birth G.S. (1982). Application of near infrared reflectance spectroscopy to cheese analysis. J. Dairy Sci., Bd..

[30] Wehling R.L., Pierce M.M. (1988). Determination of moisture in Cheddar cheese by near infrared reflectance spectroscopy. J. Assoc. Off. Anal. Chem., Bd..

[31] Blanco M., Coello J., Iturriaga H., Maspoch S. (1999). Und J. Pages, „Calibration in non-linear near infrared reflectance spectroscopy: a comparison of several methods. Anal. Chim. Acta, Bd..

[32] Næs T., Martens H. (1988). Principal component regression in NIR analysis: viewpoints, background details and selection of components. J. Chemom., Bd..

[33] Wold S., Esbensen K. (1987). Und P. Geladi, „Principal component analysis. Chemom. Intell. Lab. Syst., Bd.

[34] Haaland D.M., Thomas E.V. (1988). Partial least-squares methods for spectral analyses. 1. Relation to other quantitative calibration methods and the extraction of qualitative information. Anal. Chem., Bd.

[35] Geladi P. (1988). Notes on the history and nature of partial least squares (PLS) modelling. J. Chemom., Bd.

[36] Holcomb T.R., Hjalmarsson H., Morari M., Tyler M.L. (1997). Significance regression: a statistical approach to partial least squares. J. Chemom. J. Chemom. Soc., Bd..

[37] Bertrand D., Robert P., Launay B., Devaux M. (1987).

[38] Mutlu A.C. (2011). *u. a.*, „Prediction of wheat quality parameters using near-infrared spectroscopy and artificial neural networks. Eur. Food Res. Technol., Bd.

[39] Soto-Barajas M.C. (2013). u. a., „Prediction of the type of milk and degree of ripening in cheeses by means of artificial neural networks with data concerning fatty acids and near infrared spectroscopy. Talanta, Bd.

[40] Walczak B., Massart D. (1996). The radial basis functions—partial least squares approach as a flexible non-linear regression technique. Anal. Chim. Acta, Bd..

[41] Despagne F., Massart D.L., Chabot P. (2000). Development of a robust calibration model for nonlinear in-line process data. Anal. Chem., Bd..

[42] Marini F. (2009). Artificial neural networks in foodstuff analyses: trends and perspectives A review. Anal. Chim. Acta, Bd..

[43] Wang X., Demirci A., Graves R.E., Puri V.M. (2019). Conventional and emerging clean-in-place methods for the milking systems. Raw Milk, Elsevier.

[44] Ng W. (2019). *u. a.*, „Convolutional neural network for simultaneous prediction of several soil properties using visible/near-infrared, mid-infrared, and their combined spectra. Geoderma, Bd..

[45] Zhou L. (2022). *u. a.*, „Powdery food identification using NIR spectroscopy and extensible deep learning model. Food Bioprocess Technol., Bd..

[46] Gao J., Zhao L., Li J., Deng L., Ni J. (2021). Und Z. Han, „Aflatoxin rapid detection based on hyperspectral with 1D-convolution neural network in the pixel level. Food Chem., Bd..

[47] Chu X., Wang W., Yoon S.-C., Ni X., Heitschmidt G.W. (2017). Detection of aflatoxin B1 (AFB1) in individual maize kernels using short wave infrared (SWIR) hyperspectral imaging. Biosyst. Eng., Bd..

[48] Zheng W., Fu X., Ying Y. (2014). Spectroscopy-based food classification with extreme learning machine. Chemom. Intell. Lab. Syst., Bd..

[49] Müller-Maatsch J., Alewijn M., Wijtten M., Weesepoel Y. (2021). Detecting fraudulent additions in skimmed milk powder using a portable, hyphenated, optical multi-sensor approach in combination with one-class classification. Food Control, Bd..

[50] Sandak J., Sandak A. (2016). und R. Meder, „Assessing Trees, Wood and Derived Products with near Infrared Spectroscopy: hints and Tips. J. Infrared Spectrosc., Bd..

[51] Savitzky Abraham, Golay M.J.E. (1964). „Smoothing and differentiation of data by simplified least squares procedures. Anal. Chem., Bd..

[52] Liland K., Almøy T. (2010). und B.-H. Mevik, „Optimal Choice of Baseline Correction for Multivariate Calibration of Spectra. Appl. Spectrosc., Bd..

[53] Gan F., Ruan G. (2006). und J. Mo, „Baseline Correction by Improved Iterative Polynomial Fitting with Automatic Threshold. Chemom. Intell. Lab. Syst., Bd..

[54] Helland I.S. (1990). Partial least squares regression and statistical models. Scand. J. Stat..

[55] Akossou A.Y.J., Palm R. (2013). Impact of data structure on the estimators R-square and adjusted R-square in linear regression. Int J Math Comput, Bd..

[56] Karch J. (2020). und D. van Ravenzwaaij, „Improving on Adjusted R-squared. Collabra Psychol., Bd..

[57] Marquez L., Hill T., Worthley R. (1991). Und W. Remus, „Neural network models as an alternative to regression. Proceedings of the Twenty-Fourth Annual Hawaii International Conference on System Sciences, IEEE.

[58] Vujicic T., Matijevic T., Ljucovic J., Balota A., Sevarac Z. (2016). Comparative analysis of methods for determining number of hidden neurons in artificial neural network. Central european conference on information and intelligent systems, Faculty of Organization and Informatics Varazdin.

[59] Karsoliya S. (2012). Approximating number of hidden layer neurons in multiple hidden layer BPNN architecture. Int. J. Eng. Trends Technol., Bd..

[60] O'Shea K., Nash R. (2015).

[61] Kiranyaz S., Avci O., Abdeljaber O., Ince T., Gabbouj M. (Apr. 2021). Und D. J. Inman, „1D convolutional neural networks and applications: a survey. Mech. Syst. Signal Process., Bd..

[62] Cui C., Fearn T. (2018). Modern practical convolutional neural networks for multivariate regression: applications to NIR calibration. Chemom. Intell. Lab. Syst., Bd. 182.

[63] Zeiler M.D. (2013).

[64] Goodfellow I., Bengio Y. (2016). und A. Courville, „Deep Learning (Adaptive Computation and Machine Learning series) Illustrated Edition. Camb. Mass., S..

[65] Kingma D.P., Ba J. (2014).

[66] Valinger D. (2021). *u. a.*, „Development of ANN models based on combined UV‐vis‐NIR spectra for rapid quantification of physical and chemical properties of industrial hemp extracts. Phytochem. Anal., Bd..

